# Moonstone: a novel natural language processing system for inferring social risk from clinical narratives

**DOI:** 10.1186/s13326-019-0198-0

**Published:** 2019-04-11

**Authors:** Mike Conway, Salomeh Keyhani, Lee Christensen, Brett R. South, Marzieh Vali, Louise C. Walter, Danielle L. Mowery, Samir Abdelrahman, Wendy W. Chapman

**Affiliations:** 1 Department of Biomedical Informatics, 421 Wakara Way, University of Utah, alt Lake City, 84108 UT USA; 2 San Francisco VA Medical Center, 4150 Clement Street, San Francisco, 94121 CA USA; 3 Department of Medicine, University of California San Francisco, 505 Parnassus Ave, San Francisco, 94143 CA USA; 4 Salt Lake City VA Health Care System, 500 Foothill Drive, Salt Lake City, 84148 UT USA

**Keywords:** Natural language processing, Social determinants of health, Software

## Abstract

**Background:**

Social risk factors are important dimensions of health and are linked to access to care, quality of life, health outcomes and life expectancy. However, in the Electronic Health Record, data related to many social risk factors are primarily recorded in free-text clinical notes, rather than as more readily computable structured data, and hence cannot currently be easily incorporated into automated assessments of health. In this paper, we present *Moonstone*, a new, highly configurable rule-based clinical natural language processing system designed to automatically extract information that requires inferencing from clinical notes. Our initial use case for the tool is focused on the automatic extraction of social risk factor information — in this case, *housing situation*, *living alone*, and *social support* — from clinical notes. Nursing notes, social work notes, emergency room physician notes, primary care notes, hospital admission notes, and discharge summaries, all derived from the Veterans Health Administration, were used for algorithm development and evaluation.

**Results:**

An evaluation of Moonstone demonstrated that the system is highly accurate in extracting and classifying the three variables of interest (*housing situation*, *living alone*, and *social support*). The system achieved positive predictive value (i.e. precision) scores ranging from 0.66 (*homeless/marginally housed*) to 0.98 (*lives at home/not homeless*), accuracy scores ranging from 0.63 (*lives in facility*) to 0.95 (*lives alone*), and sensitivity (i.e. recall) scores ranging from 0.75 (*lives in facility*) to 0.97 (*lives alone*).

**Conclusions:**

The Moonstone system is — to the best of our knowledge — the first freely available, open source natural language processing system designed to extract social risk factors from clinical text with good (*lives in facility*) to excellent (*lives alone*) performance. Although developed with the social risk factor identification task in mind, Moonstone provides a powerful tool to address a range of clinical natural language processing tasks, especially those tasks that require nuanced linguistic processing in conjunction with inference capabilities.

## Background

Social risk factors are important dimensions of health and are linked to access to care, quality of life, health outcomes, life expectancy and health care utilization. Some social risk factors such as alcohol and drug abuse can be captured using administrative and laboratory data. However, data related to measures such as housing, living situation and social support are primarily recorded in free-text clinical notes, rather than as computable structured data, and hence resists easy incorporation into prediction models. In this paper, we present *Moonstone*, a new, highly configurable rule-based natural language processing (NLP) system designed to automatically extract information that require inferencing from clinical notes. The use case to which we applied Moonstone for this study is extraction of **S**ocial **D**eterminants of **H**ealth (SDOH) — specifically, *housing situation*, *living alone*, and *social support* — from clinical notes derived from the **V**eterans Health **A**dministration (VA). We chose these three variables as our focus as this information is not captured in structured fields within the VA’s administrative data, and because these domains of social risk are important to health outcomes. Building on previous rule-based clinical NLP systems [1], the Moonstone system is designed to be extensible to a range of clinical NLP tasks, especially those that involves the need for nuanced linguistic processing and inference.

### Use case: social risk factors & health

The relationship between SDOH and health outcomes is well established [2]. Lack of housing, social isolation and lack of social support are associated with higher mortality and poor health outcomes. Despite the clear relationship between SDOH and health, these metrics are not routinely used in health services and outcomes research, mainly because many of these health measures are not collected as part of routine care. Therefore, most clinical outcome studies that rely on risk adjustment do not typically utilize social risk data, and models that do incorporate SDOH data are limited to demographic information derived from structured data (e.g. race, ethnicity, rural location) [3, 4]. The importance of these metrics have been recently reinforced by the institution of Affordable Care Act penalties on hospitals with higher than average readmission rates, with the result that hospitals that care for vulnerable and disadvantaged populations are placed at financial risk. The models used by the Centers for Medicare & Medicaid Services to compare hospitals did not include measures of social risk as these factors are not available in administrative data. In recognition of the important role social factors play in health, the National Quality Forum, National Academy of Medicine, and the Department of Health and Human Services have recently emphasized the need for health care systems to identify and address social risk factors effects on patient care [5].

### Natural language processing

There are numerous NLP systems that attempt to extract clinically relevant data from unstructured clinical narratives [6]. For example, MedEx, a rule-based system designed to extract medication information — drug, dose, frequency — achieves F-scores^1^ of greater than 0.93 [7]. Similarly, MedLee (**Med**ical **L**anguage **E**xtraction and **E**ncoding System) uses a rule-based approach to extract clinically relevant information from radiology reports and discharge summaries, and has been used successfully for a number of different clinical information extraction applications (e.g. [1, 8, 9]). More recently, cTAKES (**c**linical **T**ext **A**nalysis and **K**nowledge **E**xtraction **S**ystem) utilizes open source technologies and a highly modularized system architecture in conjunction with both machine learning and rule-based methods to perform clinical information extraction tasks. The system has been used for multiple clinical NLP application domains (e.g. smoking status identification [10] and cohort identification [11]).

The NLP systems described above are designed to extract information explicitly stated in clinical text (e.g. explicit documentation of drug and alcohol use); however, a significant proportion of information regarding social context is not explicitly stated in the clinical note, but can be inferred. For example, from the statement “patient family by bedside”, it can be indirectly inferred that the patient enjoys a degree of social support (i.e. family members who visit). This inferencing process requires a degree of semantic analysis and reasoning that existing clinical NLP systems, optimized as they are for explicit information extraction, cannot easily perform. Furthermore, existing clinical NLP systems are not necessarily well suited for tasks that require the processing of highly ambiguous “everyday” words. For example, to process the sentence “patient has to stay at the VA hospital overnight because he had no one to take him home after the procedure” requires identification of everyday words, tasks, and roles, in addition to inference capabilities to arrive at the (correct) conclusion that the patient lacks social support.

Our goal with this work is to demonstrate the effectiveness of the Moonstone system’s semantic processing and inferencing capabilities by extracting and evaluating key measures of social risk — *housing situation*, *living alone*, and *social support* — from the clinical notes using NLP.

## Implementation

### Motivation

The current state of the art in automatic social risk factor analysis — as exemplified by Chen et al. [12] and Greenwald et al. [13] — utilizes a dictionary of strings or regular expressions (e.g. “patient lives alone”, “patient lacks family support”). However, there is a substantial amount of information relevant to the three variables of interest that is implicit (i.e. not stated directly) and hence is not amenable to pattern matching-based information extraction approaches. For example “social support” is often manifested in narrative notes as the interaction of patients with family members. For example, “spouse at bedside”, “accompanied to medical appointment by son”, “family member visiting regularly to help with the food and chores”, and “patient in phone contact with adult children”, are naturally understood as connoting social support. Conversely, sentences that suggest that the patient does not experience regular contact and help from family and friends imply a lack of social support. For example, if an elderly patient requires public transport to get home from a medical procedure, this can be taken as evidence — but not proof — of lack of social support. The number of possible textual instantiations of social support interactions is very large, and probably beyond the capabilities of a simple string matching approach to adequately address. Similar examples can be found for both the *housing* and *living alone* variables. For example, the statement “discharged: home with wife” implies that the patient both lives in a stable home, and does not live alone. Furthermore, the identity of the person with whom the patient lives can be indicative of whether a housing situation is stable or marginal. For example, “lives with wife” and “lives in ex-wife’s basement” both indicate that the patient does not live alone, but the latter suggests a more precarious living situation. The effort to identify implicit, indirect meaning is complicated by several factors:

1. **Inference**. The target variable is often several inference steps away from what is stated explicitly in the text. For instance, “family at bedside” literally means that family members are with the patient in the clinical care setting, which in turn implies that they are involved in the patient’s care, which connotes support.
2. **Ambiguity**. The meaning-bearing words relevant to social support are typically “everyday” high frequency words which, in contrast to medical terminology, have a high probability of appearing in contexts irrelevant to social support. For example, the word “bedside” can be used in many ways unrelated to social support (e.g. “medical equipment at the bedside”). Although potentially relevant *words* are relatively common in clinical text, relevant *sentences* appear much more sparsely, with very few documents in our corpus containing such sentences.
3. **Semantic roles**. Understanding semantic roles — i.e. who is the actor and who is the recipient of an action — is vital to sentence interpretation. For example, in the sentences “the wife helps the patient with medications” and “the patient helps the wife with medications”, only the first conveys the fact that the patient receives social support, as the patient is the direct object of the verb “helps”. Similarly, word placement can affect interpretation. For example, the sentences “he needs no help with ADL” (Activities of Daily Living) and “he has no help with ADL needs”, differ in only one word (“has”), and yet have very different meanings. NLP systems that do not consider word order, and which do not analyze the meaning and placement of modifiers, cannot reliably make such distinctions.

### Corpora & annotation

The clinical document corpus used in this study was selected with the goal of developing information extraction methods capable of automatically extracting SDOH variables relevant to 30-day readmission predictive models, with a focus on four diseases of interest (congestive heart failure, acute myocardial infarction, pneumonia, and stroke). We selected these conditions as, at the time of study initiation, they were the four medical conditions for which hospital readmission rates were publicly recorded. To create an initial cohort, we identified all VA patients aged 65 or older admitted with at least one of the four diseases of interest between January 1st 2012 and December 31st 2012. More than 21,000 patients were identified^2^. We then randomly sampled 500 patients from this cohort and extracted their associated documents — 353,889 notes in total — from the VA Corporate Data Warehouse for a period of one year prior to hospital admission. Two physicians (SK and LW) then reviewed document titles, selecting only those documents likely to contain evidence of SDOH variables. Clinical document types selected included nursing assessment, social work notes, emergency room physician notes, primary care notes, hospital admission notes, and discharge summaries. In total, 52,304 documents were selected.

Social risk factors pose a significant challenge to the creation of reliable annotated reference standards given that human annotators typically experience extreme difficulty in identifying and reliably annotating rarely documented variables. For this reason, using the corpus described above, we pre-annotated social support instances using a prototype version of Moonstone. We trained three annotators who were familiar with VA documentation practices to review a randomly selected sample of pre-annotated instances, with all disagreements between the annotators discussed until consensus was achieved. We then ran the final, trained version of Moonstone over the document set and presented disagreements between the human annotators and Moonstone to a fourth annotator (again, a nurse familiar with VA documentation practices) who was blinded as to whether the value was assigned by Moonstone or by the previous consensus of annotators. The fourth annotator selected the best variable value, which was then used as the final gold standard value.

### System description

**Fig. 1:**
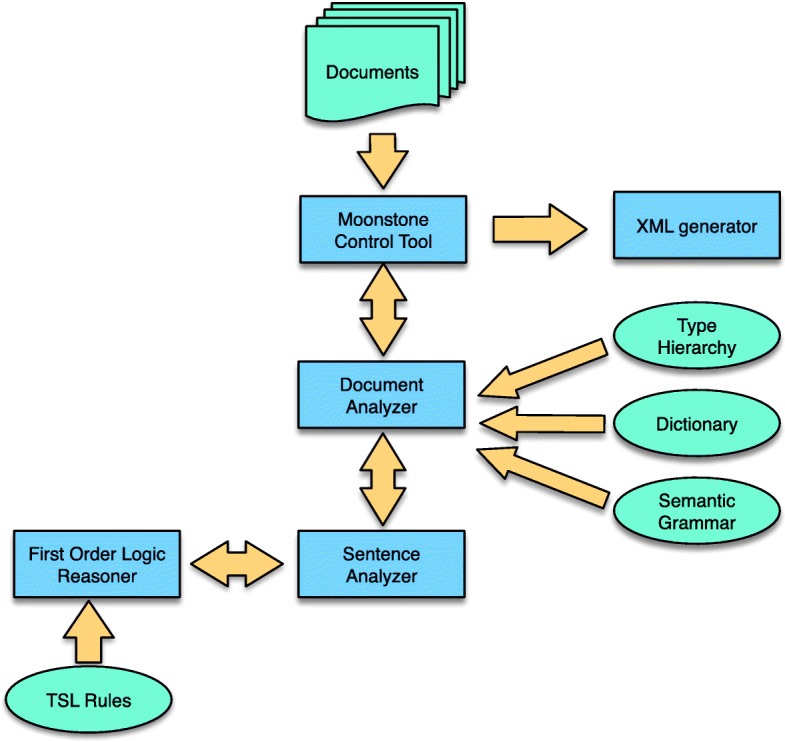
System architecture. Ovals (green) are knowledge resources, and rectangles (blue) are Moonstone components

Moonstone is an open-source, Java-based NLP system developed by the Biomedical Language Understanding Laboratory (BLULab) at the University of Utah and derived from a lineage of clinical NLP systems (ONYX [14], TOPAZ [1]) that utilize rule-based semantic analysis. The system consists of a Knowledge Base, which in turn is made up of a type hierarchy, a semantic grammar, a set of inference rules, and a word dictionary, and processing modules including a named entity recognition module, a grammatical analysis module, and an inference module. In addition, we used a tool called the Evaluation WorkBench [15] to compare two sets of human and/or machine-produced annotations over a set of documents, and display match statistics (e.g. precision, recall, accuracy, f-measure). The Evaluation WorkBench allows a human reviewer to view the annotation schema, and then view annotations highlighted within the text of the documents, thus supporting rule development and debugging. We used the WorkBench to compare Moonstone’s performance against reference standard annotations produced by human experts using the eHOST annotation tool [16]. The architecture of the Moonstone system is shown in Fig. 1, with system features described below.

Moonstone’s activities are user-controlled through its graphical control tool. A function in that tool can be used to apply Moonstone to analyze a corpus of documents. For each document, Moonstone splits that document into tokens representing words, numbers, punctuation and other symbols, attaches a dictionary definition to word tokens if available, and groups the tokens into named sections (e.g. "History of Present Illness") and sentences within those sections.

For each sentence in a document, the Moonstone grammatical parser applies the semantic grammar to analyze the tokens in that sentence, and gathers resulting concepts which are relevent to the current NLP task into a list for further processing. The grammatical parser also consults the Moonstone ontology to fire those grammar rules which contain references to semantic types in their patterns.

After the document has been parsed at the sentence level, information can be added to task-relevant annotations using first-order inference over task-specific TSL rules. After all this takes place, the task-relevant concepts are filtered to remove duplicates, then passed to the XML generator which converts them into an XML format which is readable by the eHOST annotation tool [16].

#### TSL

Moonstone includes a first-order symbolic logic called TSL (**T**yped **S**emantic **L**anguage). A first-order logic such as TSL consists of a set of symbols and syntactic rules for creating complex expressions from simpler ones, and semantic rules for determining the truth value of an expression based on the values of its components. Simple TSL expressions use a relation/argument format “(relation object1 object2...)”, and complex expressions use “and”, “or” and “not” operators to combine simple expressions into complex ones. For example, the TSL statement “(and (has-support ?patient) (not (lives-alone ?patient)))” indicates that a patient has support and does not live alone. TSL meaning predicates are stored within Moonstone’s type hierarchy and semantic grammar (described below) and assembled by the Moonstone sentence analyzer into a set of TSL statements describing the meaning of an English phrase or sentence. The collection of TSL predicates attached to an annotation object is called the object’s interpretation. In addition, each annotation is assigned a TSL constant representing a summary of the literal or inferred meaning of the sentence; for instance, the summary of “wife at bedside” is the task-relevant concept “:HAVE_SUPPORT:”.

Strengths of TSL include an ability to import static Java methods as TSL relation and function constants, and the ability to treat arbitrary Java objects as TSL object constants, thus tightly integrating TSL with the Java programming environment.

#### Knowledge base

The Moonstone Knowledge Base consists of four components: a hierarchy of semantic types implemented in TSL, a semantic grammar, a lexical dictionary, and a set of inference rules, also written in TSL.

1. **Type hierarchy.** A TSL type hierarchy is written using TSL syntax. Semantic types are standardly written in upper case with angle bracket delimiters (e.g. “<PERSON>)” and “< SOCIAL_SUPPORT >”), whereas constants denoting objects of a given type use colons as delimiters; for instance “:SOCIAL_SUPPORT:” represents an instance of social support within a TSL interpretation.
  The TSL definition statement shown in Fig. 2 declares several constants belonging to the type “<SOCIAL_SUPPORT >”, including having or lacking a companion, having or lacking care, etc.

**Fig. 2:**
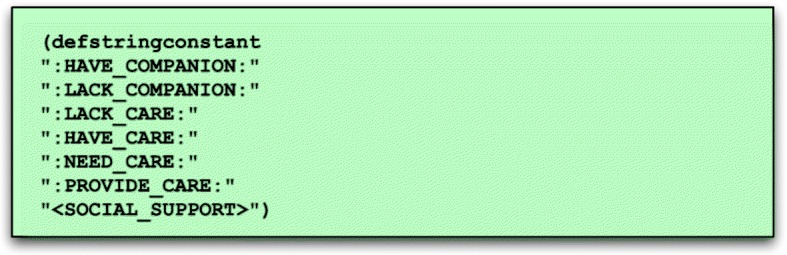
TSL example
2. **Semantic grammar.** The semantic grammar consists of a set of grammar rules containing sequences of elements that may be found in a sentence, as well as tests and constraints to validate that a rule matches a sequence of text. Semantic grammars have been used in a variety of different information extraction systems, including MedLEE [8].
  The pattern of the grammar rule shown in Fig. 3 contains only constants — i.e. symbols such as“:PERSON:” — and indicates that whenever there is a sequence of words representing a friend, family or church associate in close proximity to a non-negated phrase connoting provision of care and a phrase denoting the patient, then a new annotation is created for that sequence of words with the summary constant “:POSSIBLE_SUPPORT:”.

**Fig. 3:**
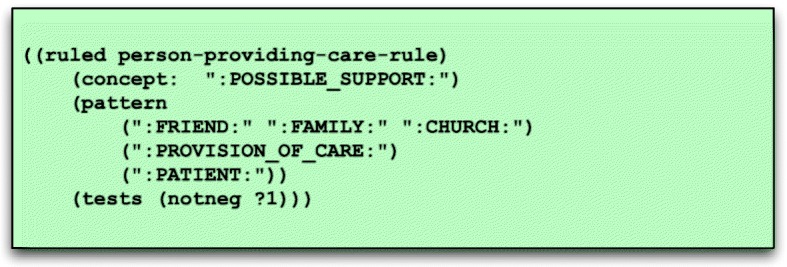
Semantic grammar rule
  Unlike most grammatical parsers, words do not need to be in left-to-right order for a match to occur, and there may be intervening words not captured in the grammar or dictionary. Not requiring a specified word order introduces some errors but makes it possible to apply a relatively small, focused grammar to identify relevant information from a document where most words are unknown and irrelevant to the NLP task at hand.
  Finally, the rule listed in Fig. 3 has one validation test: (notneg ?1), which indicates that an annotation matching the item in position 1 (?1) in the pattern list, “:PROVISION_OF_CARE:”, must not be negated. This rule, for instance, would not match “he receives no help from his family”.
3. **Lexical dictionary.** Moonstone uses an optional dictionary of words and phrases, extracted from the UMLS (**U**nified **M**edical **L**anguage **S**ystem) Metathesaurus [17] that may be suitable for tasks that require the use of extensive terminological resources (e.g. drug name extraction). However, since the relevant words in the VA readmission tasks are predominantly common words that do not appear in specialized vocabularies such as SNOMED (**S**ystematized **No**menclature of **Med**icine) or RxNorm, word-level grammar rules are used in place of a dictionary in this project. For example, the word-level grammar rule shown in Fig. 4 includes words and phrases such as “husband”, “wife”, and “significant other”, with these words and phrases associated with a normalizing constant — in this case “:SPOUSE:” — which is output when the words or phrases are encountered.

**Fig. 4:**
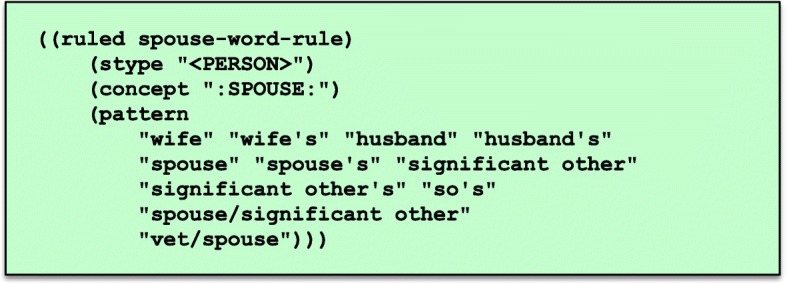
Word-level grammar rule mapping phrases to normalizing constant “:SPOUSE:”. Note that these rules were defined for the US healthcare system and hence may not prove appropriate for non-US contexts
4. **TSL inference rules.** TSL includes an inference engine which is invoked by the semantic grammar analysis module to assign interpretations to sentences when the semantic grammar does not provide sufficient information to do so. The TSL rule shown in Fig. 5 determines that the subject of a sentence represents a friend or family member, and that the sentence contains a non-negated reference to the concept “LIVE_AT_HOME”. This rule will match sentences such as “Grandson currently living with the patient in his apartment”, and infer that the patient does not live alone.

**Fig. 5:**
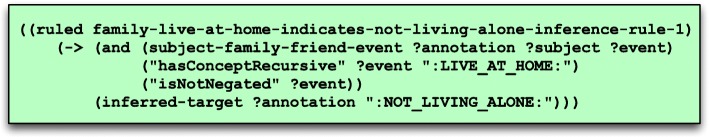
TSL rule used to augment grammatical analysis

#### Named entity module

Moonstone’s Named Entity Recognition (NER) module is not shown in Figure 1. The NER module uses a fast, computationally efficient algorithm inspired by the IndexFinder NER tool, a widely used resource in clinical NLP [18]. The NER module applies the dictionary to a document to identify word and phrase-level medical expressions prior to the application of the semantic grammar. However, since the focus of this project is social risk factors, this module is not used.

#### Grammar analysis module

**Fig. 6:**
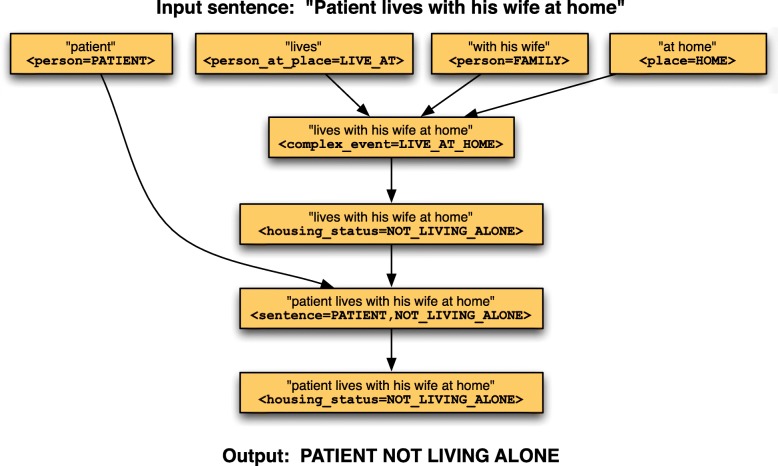
Parse tree for “patient lives with his wife at home”

The grammar analysis module applies a semantic grammar to each sentence in a document, and outputs a set of TSL expressions representing the sentence’s interpretation. The module uses a variation of the CYK context-free grammar parsing algorithm [19], to perform bottom up text analysis. Beginning with word-level annotations, the module searches for sequences of annotations that match grammar rule patterns. An annotation may match a rule pattern element based on its summary constant (e.g. “:PATIENT:”) or its semantic type (e.g. “<PERSON>”). Figure 6 shows the complete parse tree generated for the sentence “the patient lives with his wife at home”.

Note that with a large or complex grammar there will often be many alternative parse trees, often with different interpretations. Moonstone selects the most likely trees based on probabilities learned during the training process, and on the prevalence of task-relevant concepts such as “:NOT_LIVING_ALONE” and “:HAVE_SUPPORT:”.

#### TSL inference module

TSL includes two inference engines, one that uses *forward-chaining* inference (similar to widely used inference engines like Drools [20]), and one that uses *backward chaining* inference (similar to Prolog [21])^3^. In addition to interacting with the grammar module as described earlier, the inference module can be applied after grammatical analysis to identify implications of the sentence-level interpretations. This functionality can be used to drive such processes as storing relevant information to a database, sending alerts to clinicians, etc.

#### Training module

One of the most challenging aspects in the development of any tool capable of symbolic knowledge processing is the development of supporting Knowledge Bases, including lexicons, ontologies and grammars. On the basis of our experience working with VA clinical texts, writing and debugging useful semantic grammars by hand is time consuming, frustrating and prone to error. With this in mind, we have developed a training tool to aid in expanding a set of abstract grammar rules into a larger set of rules tailored for a specific NLP task. We begin with a limited set of basic, domain-neutral rules potentially applicable to a large set of NLP projects. The training tools permit a human trainer to apply Moonstone to a corpus of training documents. For each structurally correct parse tree, the trainer selects from a menu the intended TSL summary constant for that tree. The training tool then creates a new specialized grammar rule based on the structure of the abstract rule, containing the semantic constants in the parse tree as the new pattern, and assigning the user-selected constant an interpretation.

## Results and discussion

In this paper, we introduce Moonstone, a system designed to support the automatic extraction and classification of information from clinical notes, including information that requires inferencing from lower-level concepts. The use case to which we applied Moonstone is that of identifying mentions of social risk factors (*housing situation*, *living alone*, and *social support*). When applied to a blind test set with a manually annotated reference standard^4^, Moonstone’s results ranged from good to excellent, with positive predictive value (i.e. precision) scores ranging from 0.66 (*homeless/marginally housed*) to 0.98 (*lives at home/not homeless*), accuracy scores ranging from 0.63 (*lives in facility*) to 0.95 (*lives alone*), and sensitivity (i.e. recall) scores ranging from 0.75 (*lives in facility*) to 0.97 (*lives alone*) (see Table 1).

**Table 1:** Mention level classification results

**Variable**	TP^a^	FP^b^	FN^c^	Sen^d^	NPV^e^	PPV^f^	Acc^g^	F-score^h^
Housing Situation								
Homeless/marginally housed	79	39	11	0.87	0.38	0.66	0.63	0.75
Lives at home/not homeless	6382	111	335	0.95	0.02	0.98	0.93	0.96
Lives in a facility	426	131	141	0.75	0.26	0.76	0.63	0.75
Living Alone								
Does not live alone	1329	123	88	0.93	0.11	0.91	0.86	0.92
Lives alone	710	19	17	0.97	0.05	0.97	0.95	0.97
Social Support								
Has social support	5174	525	337	0.93	0.21	0.90	0.85	0.92
No social support	220	59	19	0.92	0.78	0.78	0.78	0.84

^a^True positive

^b^False positive

^c^False negative

^d^Sensitivity (i.e. recall)

^e^Negative predictive value

^f^Positive predictive value (i.e. precision)

^g^Accuracy

^h^F-score (harmonic mean of positive predictive value and sensitivity)

For some categories performance was good but not excellent (*homeless/marginally housed* and *lives in facility* both achieved 0.63 accuracy). We suspect that performance was lower for these categories, because they require *inference* from the text by Moonstone, rather than being explicitly stated in the text. Consider the example of the *lives alone* category. It is common for clinicians to directly ask, and explicitly document this information, making Moonstone’s task easier. Another possibly salient factor is that some of the lower performing variables occur with relatively low frequency in our corpus. For example *homeless/marginally housed* has 79 True Positives, whereas *lives at home/not homeless* has 6382.

To estimate the degree to which Moonstone utilizes inference to map from text to domain concepts, we identified those grammar rules that directly produce concepts, as opposed to rules that produce lower and intermediate-level interpretations en route to concepts, then we re-ran Moonstone on the evaluation corpus and calculated the percentage of times the “direct” rules were used. Some rules, like those that directly map the word “homeless” to the concept Homeless/Marginally Housed, appeared frequently in analyses producing those concepts. Others, like the rule recognizing phrases like “supportive family” and “good system of support” as indicators of Social Support were used in fewer than 5% of analyses recognizing support. The remaining 95% of phrases required Moonstone’s recognition of many types of interactions involving family and friends as indicative of support. Table 2 presents percentages that reflect the proportion of direct grammar rule involvement in Moonstone’s analyses, and thus illustrate (at least approximately) the proportion of indirect inference used in those analyses, hence indicating the importance of Moonstone’s inferencing capabilities for complex NLP tasks.

**Table 2:** Proportion of direct grammar rules used per category

Concept	Proportion of direct grammar rules utilized
Lives Alone	25.0%
Does Not Live Alone	0.6%
Has Social Support	4.8%
Lacks Social Support	20.0%
Not homeless/lives at home	5.8%
Homeless/marginally housed	80.5%
Lives In Facility (e.g. nursing home)	0.0%

One key challenge associated with developing clinical NLP for VA data lies in the highly templated (i.e. semi-structured) nature of VA clinical notes [22], including check boxes and structured question and answer templates. For example, homelessness can be represented in clinical narratives in various ways (“pt not homeless”, “HOMELESS:1”, “homeless:y”). Automatically distinguishing between free-text, structured and semi-structured areas of the note remains a significant challenge [23]. A further challenge is presented by the widespread use of highly “telegraphic” language in clinical notes, including often idiosyncratic abbreviations and truncations, missing function words, ambiguity, and misspellings. These challenges demand the creation of special purpose NLP tools capable of both distinguishing between semi-structured data and narrative text, and processing text that is frequently ungrammatical.

Moonstone uses a linguistically-oriented rule-based approach. This is in contrast to widely used approaches in the general domain NLP community, where machine learning — in particular, modern neural network based machine learning [24] — is frequently used. We adopted a rule-based approach for two reasons. First, annotated data necessary to both train and evaluate a machine learning algorithm is very expensive to obtain in the clinical domain. Rule-based methods allow for validation and evaluation of algorithms using a much smaller — and hence less expensive — data set. Second, machine learning based NLP algorithms are somewhat opaque (i.e. the *reason* that a particular classification is made is often a function of various non-pellucid numerical weights within the algorithm). Rule-based methods are typically substantially more perspicuous, given that the *reasons* for a particular classification decision can be clearly articulated. However, the perspicuousness of Moonstone’s rule-based inferencing capabilities is accompanied by costs, including relative computational inefficiency (i.e. Moonstone may not be suitable for processing millions of notes), and the need to manually construct complex Knowledge Bases.

In future work, we intend to utilize the Moonstone mention-level social support classifier described in this paper as input to a machine learning based patient-level classifier, with the resulting patient-level classification variables integrated into a 30-day readmission model, allowing us to formally evaluate the contribution of social risk factor variables to 30-day readmission algorithm performance.

## Conclusions

The Moonstone system is an open-source NLP system designed for clinical NLP tasks that require nuanced linguistic processing and inferencing capabilities. The system achieved excellent results in the challenging task of extracting SDOH variables from VA clinical notes. We are currently applying the rules developed on VA text to non-VA notes at three locations in order to determine the extent to which the system is portable between sites. Although developed with the social risk factor identification task in mind, Moonstone provides a powerful tool to address a range of clinical NLP tasks, especially those tasks that require nuanced linguistic processing in conjunction with inferencing capabilities.

## Availability and requirements

**Project name**: Moonstone

**Project homepage**: https://github.com/Blulab-Utah/VAReadmissionMoonstone

**OS**: Java/Multi-platform

**Programming language**: Java

**Other requirements**: No other requirements

**License**: Apache 2

**Any restrictions on use by non-academics?**: No restrictions

## Abbreviations

ADL: Activities of daily living
BLULab: Biomedical language understanding laboratory
cTAKES: Clinical text analysis and knowledge extraction
ICD-9: International classification of diseases, ninth revision
NER: Named entity recognition
NLP: Natural language processing
SDOH: Social determinants of health
SNOMED: Systematized nomenclature of medicine
TSL: Typed semantic language
UMLS: Unified medical language system
VA: Veterans administration

## Footnotes

1 *F-score* is the harmonic mean between positive predictive value (i.e. precision) and sensitivity (i.e. recall)

2 We limited the sample to those individuals 65 years of age and older as this would allow for comparison with readmission outcome ascertained using both VA and Medicaid data.

3 Forward chaining is an inference method which begins with a set of declared facts, then iteratively applies a set of rules to identify all possible conclusions. In contrast, backward chaining begins with the conclusion and works backwards to identify supporting facts.

4 20% of the annotated data was held out for evaluation.
